# Enhancing Subjective Wellbeing in Older Individuals with Amnestic Mild Cognitive Impairment: A Randomized Trial of a Positive Psychology Intervention

**DOI:** 10.3390/bs13100838

**Published:** 2023-10-13

**Authors:** Konstantina Tsiflikioti, Despoina Moraitou, Christos Pezirkianidis, Georgia Papantoniou, Maria Sofologi, Georgios A. Kougioumtzis, Magdalini Tsolaki

**Affiliations:** 1 Faculty of Medicine, Leiden University, 2333 ZA Leiden, The Netherlands; 2 School of Psychology, Faculty of Philosophy, Aristotle University, 54124 Thessaloniki, Greece; 3 Laboratory of Psychology, Department of Cognition, Brain and Behavior, School of Psychology, Faculty of Philosophy, Aristotle University, 54124 Thessaloniki, Greece; 4 Laboratory of Neurodegenerative Diseases, Center for Interdisciplinary Research and Innovation (CIRI-AUTH), Balkan Center, Aristotle University, 10th km Thessaloniki-Thermi, 54124 Thessaloniki, Greece; tsolakim@auth.gr; 5 Day Center “Greek Association of Alzheimer’s Disease and Related Disorders (GAADRD)”, 54643 Thessaloniki, Greece; 6 Interdisciplinary Mental Health Centre of the Armed Forces Board of Members, Hellenic Association of Positive Psychology, Panteion University, 17671 Athens, Greece; pezir@panteion.gr; 7 Laboratory of Psychology, Department of Early Childhood Education, School of Education, University of Ioannina, 45110 Ioannina, Greece; gpapanto@uoi.gr (G.P.); m.sofologi@uoi.gr (M.S.); 8 Institute of Humanities and Social Sciences, University Research Centre of Ioannina (URCI), 45110 Ioannina, Greece; 9 Department of Turkish Studies and Modern Asian Studies, Faculty of Economic and Political Sciences, National and Kapodistrian University of Athens, 15772 Athens, Greece; gkougioum@ppp.uoa.gr; 10 Department of Psychology, School of Health Sciences, Neapolis University, 8042 Pafos, Cyprus; 11 School of Social Sciences, Hellenic Open University, 26335 Patras, Greece; 12 1st Department of Neurology, Medical School, Aristotle University of Thessaloniki (AUTh), 54124 Thessaloniki, Greece

**Keywords:** older individuals, (amnestic) mild cognitive impairment, positive psychology, intervention, well-being

## Abstract

Objectives: This pilot study aims to explore the potential of a positive psychology intervention (PPI) in enhancing the subjective well-being of older individuals with amnestic mild cognitive impairment (MCI), a precursor to dementia. Design and Setting: A randomized trial was conducted, initially recruiting 51 participants aged 65 and above from the Greek Association of Alzheimer’s Disease and Related Disorders in Thessaloniki, Greece. The study employed a control-experimental group setup. To ensure randomization, each participant was assigned a unique number, and a random number generator was used for group allocation. Participants: A total of 41 eligible participants with amnestic mild cognitive impairment (MCI) were included in the study after screening. Intervention: The intervention consisted of a 3-week positive psychology program (PPI) where the PERMA Profiler questionnaire was administered at three intervals: pre-intervention, post-intervention, and one month after completion. Main Outcome Measures: The subjective well-being of participants. Results: The analysis, conducted mainly through mixed-measures ANOVAs, supported the study’s hypotheses, revealing that the 3-week PPI led to increased PERMA model scores and overall well-being, which persisted even after one month. Conversely, non-participants experienced declines in most domains except for Positive Emotion and Meaning, which demonstrated improvement and recovery during follow-up. Conclusions: These findings suggest the potential of PPI in enhancing the subjective well-being of older adults with amnestic MCI, with implications for addressing dementia-related challenges. Further investigation is warranted to pinpoint PPI effects on MCI and tailor interventions for improved subjective well-being.

## 1. Introduction: Background and Objectives

### 1.1. Positive Psychology: A Paradigm Shift in Understanding Well-Being and Human Flourishing 

Positive psychology emerged in response to a historical focus in psychology primarily on pathology, which inadvertently neglected the holistic development of individuals and the thriving of communities before the 2000s [1]. This paradigm shift in the field emphasizes not only addressing weaknesses and problems but also cultivating strengths and positive aspects of life [1]. Positive psychology complements traditional psychology by offering a broader perspective on human experiences and well-being [2]. Its aim is to augment, not replace, existing knowledge about human suffering, weakness, and disorder, striving for a comprehensive understanding of the human experience [3]. The central focus of positive psychology lies in enhancing subjective well-being, encompassing positive emotions, engagement, and meaning, often through psychological interventions, which, in turn, lead to a range of benefits across social, intellectual, and physical domains, ultimately improving an individual’s quality of life and objective well-being [4,5].

However, it is important to distinguish between subjective and objective well-being. More specifically, subjective well-being refers to an individual’s self-assessment of life satisfaction and happiness [6], encompassing emotional experiences, purpose, and fulfillment. It focuses on internal perceptions and can vary across individuals [7,8,9,10]. Objective well-being, on the other hand, measures factors such as income, education, health, and social relationships considering, in other words, basically external factors [9,10,11].

In addition to those, there are other facets of well-being, specifically eudaimonic and hedonic well-being, which encompass aspects of psychological well-being. In brief, hedonic well-being focuses on the pursuit of pleasure and avoidance of pain, emphasizing subjective experiences of happiness and satisfaction, while eudemonic well-being centers on the pursuit of meaning, purpose, and self-actualization [12]. Psychological well-being, on the other hand, encompasses a broader scope of overall mental well-being. It includes various dimensions such as positive emotions, engagement, meaning, positive relationships, and accomplishment. While eudemonic and hedonic well-being are aspects of psychological well-being, the latter encompasses a wider range of dimensions that contribute to an individual’s overall psychological functioning and quality of life [13]. Older individuals facing a range of health conditions frequently encounter heightened levels of depressive emotions in conjunction with diminished hedonic and eudaimonic well-being [14].

Empirical evidence indicates that certain interventions within the realm of positive psychology have demonstrated enduring effects in augmenting happiness and reducing symptoms of depression [3]. More specifically, for example, it has been demonstrated that interventions centered on gratitude increase levels of positive affect, duration and quantity of sleep, optimism, and a sense of connectivity to others [15]. Grateful thinking motivates individuals to actively recognize and savor positive life experiences and situations, enabling them to derive heightened levels of satisfaction and pleasure from their surroundings [16]. Another example is reminiscence interventions that demonstrate a wide range of effects on various outcomes, exhibiting therapeutic and preventive benefits such as those observed in other commonly used positive psychology interventions (PPIs). Reminiscence refers to the process of reflecting upon and sharing personal experiences from the past that hold personal significance [17]. 

Overall, multiple meta-analytic studies have consistently demonstrated the positive effects of PPIs across diverse domains, such as well-being, depression, anxiety, and stress. These interventions have exhibited effect sizes ranging from small to medium at both post-treatment and follow-up stages and notably, the domains of well-being and depression exhibited the largest effect sizes [18,19,20,21]. However, it is important to note that these effects were predominantly observed in non-clinical samples, encompassing a comprehensive array of PPIs [18,19,20,21].

### 1.2. The Growing Burden of Dementia and Mild Cognitive Impairment: Implications for Aging Populations and Quality of Life

The population of individuals aged 60 years and older is steadily growing, and so is the prevalence of dementias which is projected to rise from its current estimate of 1 million to 1.6 million in the upcoming decades [22]. Dementias are syndromes that arise from various diseases, progressively damaging the brain and destroying nerve cells. This ultimately leads to a serious decline in cognitive function, extending beyond the normal effects of biological aging. Dementias exerts physical, psychological, social, and economic consequences, affecting not only individuals living with the condition but also their caregivers, families, and society. Currently, dementias hold the position of the seventh leading cause of death worldwide and represent a substantial factor in the prevalence of disability and dependence among older individuals [23].

Mild cognitive impairment (MCI) serves as a prodromal stage preceding the onset of dementia and describes the intermediate stage between the cognitive changes associated with normal aging and the early signs of dementia, where individuals experience noticeable cognitive decline beyond what is considered typical for their age [24]. MCI imposes a significant burden, leading to a decline in quality of life and a negative impact on psychological well-being [25]. Compared to individuals without any cognitive impairments, those with MCI demonstrated lower levels of social support, self-esteem, life satisfaction, positive affect, optimism, and hope, while exhibiting higher levels of negative affect demonstrating the need to intervene [26]. 

MCI can be classified into two primary subtypes: amnestic MCI, which is the most common form of MCI and is characterized by memory impairment as the predominant feature, and non-amnestic MCI, which primarily affects other cognitive functions beyond memory, such as attention, language, executive functions, etc. Individuals with amnestic MCI often experience difficulties in remembering recent events, recalling information, or retaining new learning [27,28] and this form of MCI is commonly associated with an increased risk of developing Alzheimer’s disease [29]. On the other hand, non-amnestic MCI encompasses a broader range of cognitive deficits, and the underlying causes can vary. It may be associated with other neurodegenerative disorders, such as vascular dementia or frontotemporal dementia, depending on the specific cognitive impairments present [27,28].

### 1.3. Positive Psychology Interventions: Exploring Efficacy and Potential for Enhancing Well-Being in Individuals with Mild Cognitive Impairment

In most cases, researchers tend to implement single-type PPIs, as opposed to multi-element programs. In the former scenario, these interventions encompass a wide spectrum of practices, including but not limited to gratitude exercises, forgiveness exercises, acts of kindness, meaning-making exercises, savoring techniques, relationship-strengthening activities, the identification and utilization of signature strengths, volunteering experiences, and more. Conversely, in the latter case, PPI programs typically incorporate an amalgamation of approximately five different types of practices. Moreover, the delivery of PPIs predominantly occurs within a group setting, constituting 42.36% of cases, whereas individual-level delivery accounts for a more limited proportion at 12.10%. Furthermore, while it is common for PPIs to be administered online with the guidance of a therapist or coach, the primary mode of delivery remains in-person. Additionally, the average duration of PPIs spans 6.35 weeks, equivalent to approximately 10.41 sessions on average [30].

Research shows that applying PPIs and techniques effectively increases subjective well-being and mental health levels. More specifically, positive contributions have been found in terms of depressive symptoms, psychological well-being, anxiety and stress, quality of life, and life satisfaction [18,19,20,21].

The current body of literature concerning the investigation of positive psychology in individuals with MCI is limited. Most of the research into the topic thus far has predominantly concentrated on individuals with dementia, revealing positive outcomes of PPIs in promoting subjective well-being. Notably, these interventions have been associated with various benefits, such as the reduction in sadness and depression, an enhancement of pleasure and satisfaction [31], improved quality of life, better subjective sleep quality [32], increased positive affect, and improved communication skills [33]. However, there is a need for further research to expand our understanding of the specific effects of PPIs on individuals with MCI in its different subtypes, and to examine the potential effects of PPIs on people who are still functional and relatively independent in their everyday life. 

### 1.4. Aim and Hypotheses of the Current Study

Given the accumulated evidence suggesting the favorable effects of positive psychological factors on various health outcomes, it is imperative to explore the potential link between positive psychological factors and the management of MCI. The present pilot study aimed to determine whether a PPI improves the subjective well-being of older individuals with amnestic MCI, a type of MCI that can progress to the most common type of dementia, namely, Alzheimer’s disease dementia. To address this, we formulated two hypotheses. Hypothesis 1 posits that the Positive Psychology Intervention (PPI) will increase the subjective well-being of individuals diagnosed with amnestic Mild Cognitive Impairment (MCI). Hypothesis 2 suggests that subjective well-being in patients diagnosed with amnestic MCI will persist at elevated levels one month after the conclusion of the PPI.

## 2. Materials and Methods

### 2.1. Study Design and Participants 

The pilot study was structured as a randomized trial, with randomization achieved by assigning a distinct identifier to each participant and employing a computer-based random number generator for group allocation (see Appendix A).

To be eligible for the study, participants needed to be 65 years or older and have a diagnosis of amnestic MCI. At this point, it should be noted that they all participate in non-pharmaceutical cognitive interventions and therapy. The exclusion criteria for this study were as follows: (a) serious psychiatric or neurological disorders, (b) cancer in the last 5 years, (c) serious cardiovascular diseases, (d) substance abuse or any type of dependence, and (e) concurrent participation in similar interventions to ensure the intervention’s effects are not confounded by multiple interventions. 

**Figure 1:**
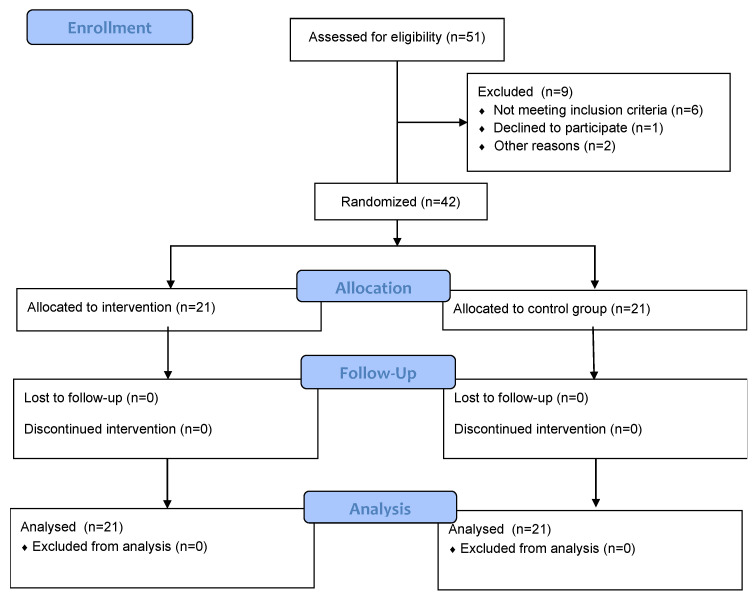
Flow chart of participants recruitment and randomization.

Following a power analysis conducted with G*Power [34], it was determined that a total sample size of 158 participants would be required to detect an effect size of η^2^ = 0.25, using a significance level of alpha = 0.05, and achieve a power of 0.80. Regrettably, due to availability constraints, achieving this recommended sample size was not feasible. More specifically, a total of 51 participants between the ages of 65 and 90 were recruited from the Greek Association of Alzheimer’s Disease and Related Disorders (Alzheimer Hellas) in Thessaloniki, Greece. Among them, nine were excluded from the study; six of them did not meet inclusion criteria, one declined to participate, and two could not participate due to lack of time (see Figure 1).

Finally, 42 participants, 37 women (88.1%) and 5 men (11.9%), with a mean age of 72.17 years (S.D. = 4.6) were randomly assigned to either the control or the experimental group. Of these five had completed only primary school, 17 had completed secondary school and the rest had finished higher education. The groups did not differ significantly in gender χ^2^ (1) = 0.227, *p* > 0.05, age F (1) = 0.573, *p* > 0.05, or educational level χ^2^ (1) = 9.29, *p* > 0.05 (see Table 1).

#### 2.1.1. Procedure 

Individuals with amnestic MCI were included in the study based on DSM-5 criteria for Mild Neurocognitive Disorders [35]. Their diagnosis involved a comprehensive evaluation, including neurological examination, neuropsychological and neuropsychiatric assessments, neuroimaging, and blood tests. Specialized healthcare professionals from Alzheimer’s Hellas reached a consensus on the diagnosis. Inclusion criteria comprised meeting the diagnosis of Minor Neurocognitive Disorders [35], achieving a minimum Mini-Mental State Examination (MMSE) total score of 24 [36,37], corresponding to stage 3 on the Global Deterioration Scale [38], and exhibiting performance at least 1.5 standard deviations below the normal mean in the memory domain, adjusted for age and education.

The PERMA Profiler (PERMA-23) questionnaire was sent either through email or the application Viber depending on the preference of the participant. For those who had difficulty accessing the above applications, a third option was given to complete the questionnaire by phone. All study participants completed the questionnaire three times: prior to the initiation of the intervention for the experimental group, immediately after its completion, and one month later (follow-up). The experimental group was divided into three subgroups, each consisting of seven individuals to facilitate the process of the intervention. The intervention lasted for three weeks, comprising a total of six online sessions, with two sessions per subgroup per week lasting 45–60 min each. Each session involved a different positive psychology exercise and some sessions also involved homework. To ensure adherence to the homework participants were informed that at the end of the procedure, the homework would be collected by the researcher. Additionally, the control group only maintained their regular participation in online cognitive therapy without any supplementary intervention or researcher involvement. They actively participated in online sessions, conducted roughly twice a week, each lasting approximately 45–60 min. These sessions were specifically designed to enhance their cognitive abilities, addressing aspects such as memory, orientation, attention, language skills and other cognitive domains.

#### 2.1.2. Blinding

In this study, a single-blind method was employed, where participants were unaware of their assigned intervention, while the researcher remained informed.

#### 2.1.3. Ethics

Participation in the study was voluntary, and no incentives were provided. Participants were informed about the study’s purpose and the sourcing of their demographic and diagnostic data from Alzheimer’s Hellas. The study protocol was approved by the Scientific and Bioethics Committee of Alzheimer’s Hellas (protocol number 87/09-03-2023), following the guidelines of the General Data Protection Regulation (EU) 2016/679. The study adhered to the principles of the Helsinki Declaration, protecting the rights and well-being of participants.

### 2.2. Instruments

#### 2.2.1. PERMA Profiler (PERMA-23)

The PERMA Profiler (PERMA-23) questionnaire is a validated measurement tool specifically developed to assess various aspects of subjective well-being. It is grounded in the PERMA model, which encompasses 5 fundamental dimensions of well-being: Positive emotion, Engagement, Relationships, Meaning, and Accomplishment, each measured with three items. Additionally, the questionnaire includes supplementary items, but they were not used in this study. Furthermore, an overall well-being score can be calculated by averaging the dimension scores and a single happiness item. The questionnaire utilizes an 11-point Likert-type scale, where respondents rate their level of agreement or frequency on a scale ranging from 0 to 10. The adaptation of the PERMA Profiler has been established through a validation study conducted on a Greek sample, confirming the questionnaire’s structure, and demonstrating acceptable internal consistency, stability, and validity. More specifically, the reliability coefficients were: (a) positive emotions: α = 0.83, (b) engagement: α = 0.56, (c) relationship: α = 0.74, (d) meaning: α = 0.78, (e) accomplishment: α = 0.72, (f) overall well-being: α = 0.91 [39]. It should be highlighted that the present study focused entirely on the overall well-being score and the 5 core dimensions of the PERMA model.

#### 2.2.2. Positive Psychology Intervention (PPI)

The PPI employed in this study was based on previous empirical research. Specifically, the sessions involving gratitude and character strengths drew heavily from the work of Seligman et al. [3]. Gratitude was conceptualized as the awareness of and appreciation for positive occurrences, while character strengths encompassed six virtues: wisdom and knowledge, courage, humanity, justice, temperance, and transcendence, which incorporate additional admirable traits. Additionally, positive reminiscence was defined as the act of recalling positive memories, serving as a constructive tool to enhance present awareness and provide a sense of perspective. This session drew inspiration from the work of Bryant, Smart, and King [40]. Kindness, another session component, was defined as performing helpful acts for others, demonstrating caring skills, and offering unsolicited assistance and it was based on Macfarlane’s research [41]. Lastly, the humor session was derived from a variation of the “three good things” exercise, focusing instead on “three funny things” made by Gander et. al. This adaptation allowed participants to recall and reflect on humorous experiences as a means to foster positive emotions and well-being (see Table 2) [42].

## 3. Statistical Analysis

The IBM SPSS Statistics version 27 software package was used for data processing [43]. Chi-squares and one-way ANOVA were used to determine if there were significant differences in demographic data across the two groups. Subsequently, mixed-measures ANOVAs were utilized to analyze the study variables, aiming to investigate the impact of the intervention, the effect of time of measurement, and potential interaction effects. The objective of these analyses was to evaluate and compare the performance of both groups, while simultaneously assessing the performance of each group across three different time points. Ultimately, in response to the G*power analysis indicating the inadequacy of our sample size, we opted for non-parametric Kruskal–Wallis analyses, known for their robustness in the presence of smaller sample sizes.

### 3.1. Results

While the primary focus of the study centered around the overall well-being score, it is worth mentioning that the relatively small sample size prompted the examination of the five dimensions of the PERMA model. For each one of the dimensions of the PERMA model plus the overall well-being in the three different time points, the descriptive statistics of the demographic variables for the intervention and control groups are summarized in Table 3.

#### 3.1.1. Overall Wellbeing

**Figure 2:**
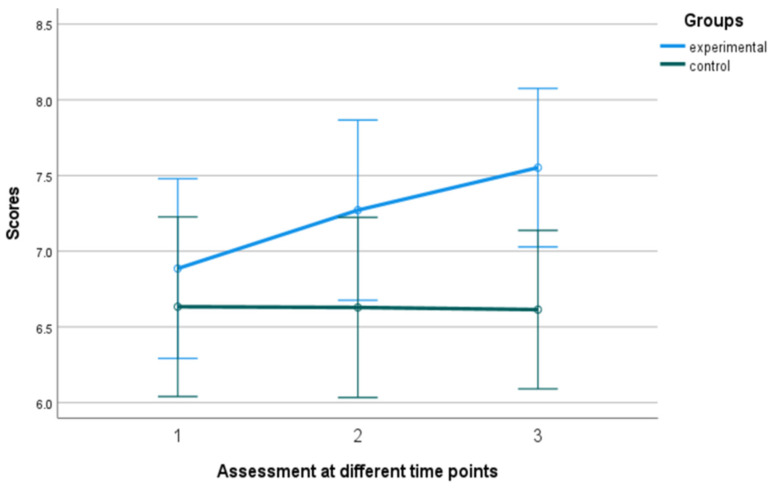
Subjective well-being in the experimental and control group. Numbers 1, 2, and 3 explain the 3 assessment time points. Number 1 indicates before the PPI, number 2 indicates after the PPI and number 3 indicates the follow-up assessment.

**Figure 3:**
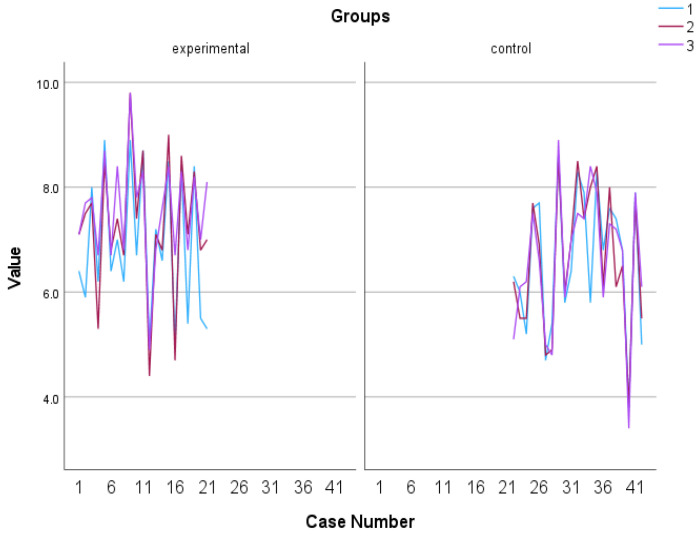
Individual scores in subjective well-being. Numbers 1, 2, and 3 explain the 3 assessment time points. Number 1 indicates before the PPI, number 2 indicates after the PPI and number 3 indicates the follow-up assessment.

**Figure 4:**
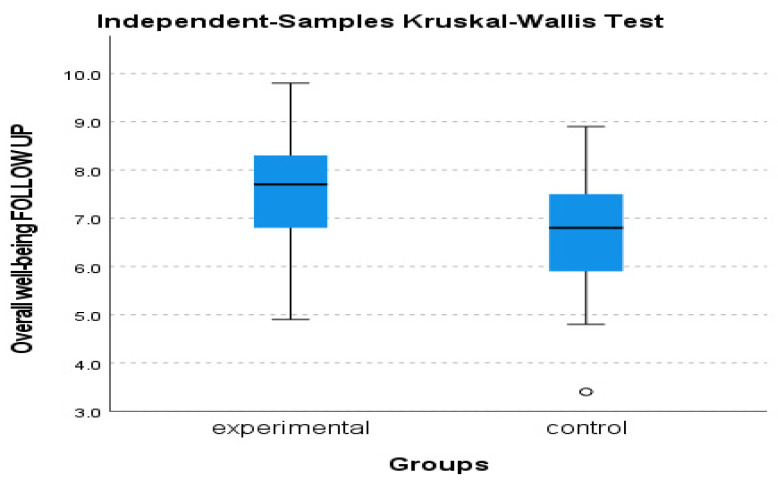
Subjective well-being follow-up assessment of experimental and control group.

A mixed-design ANOVA with a 2 × 3 arrangement was conducted, using the group as the between-subjects factor and the time of assessment as the within-subjects factor. Regarding the overall well-being, a significant tendency in the group x time of assessment effect was observed, F (2, 39) = 3.199, *p* = 0.052, η^2^ = 0.141. The control group exhibited a relatively stable performance, displaying minimal fluctuations over time. In contrast, the experimental group demonstrated a consistent and substantial upward trajectory, exhibiting significant and rapid improvement (see Figure 2 and Figure 3). The Kruskal–Wallis test revealed a significant difference solely in the follow-up assessment of the variable, with a η^2^ = 0.513 and *p* = 0.023 (see Figure 4).

#### 3.1.2. Positive Emotion and Relationship

**Figure 5:**
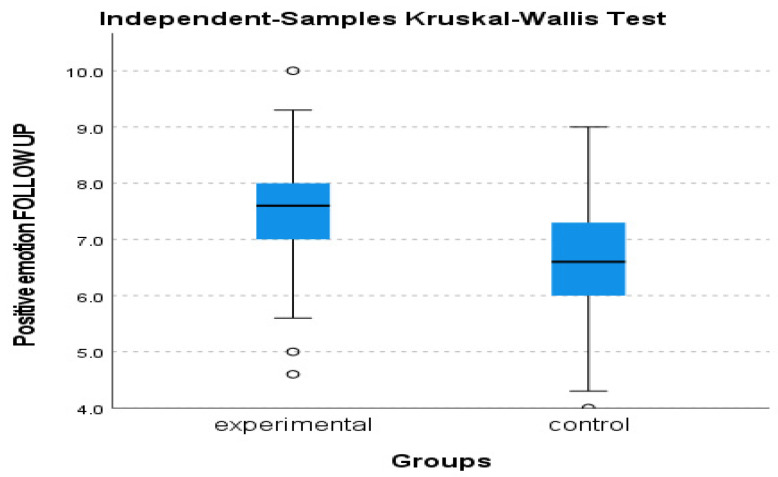
Positive Emotion follow-up assessment of experimental and control group.

Regarding the dimensions of Positive Emotion and Relationship, no significant results were found in terms of the ANOVAs analyses, however, Kruskal–Wallis revealed a significant statistical difference between groups in the positive emotion follow-up assessment, with an η^2^ = 0.489 and *p* = 0.027 (see Figure 5).

#### 3.1.3. Engagement

**Figure 6:**
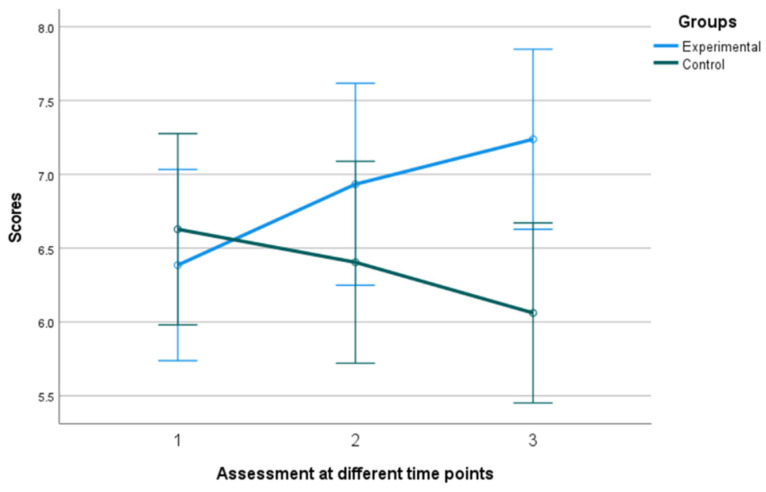
Performance of groups in Engagement in the 3 assessment time points. Numbers 1, 2, and 3 explain the 3 assessment time points. Number 1 indicates before the PPI, number 2 indicates after the PPI and number 3 indicates the follow-up assessment.

**Figure 7:**
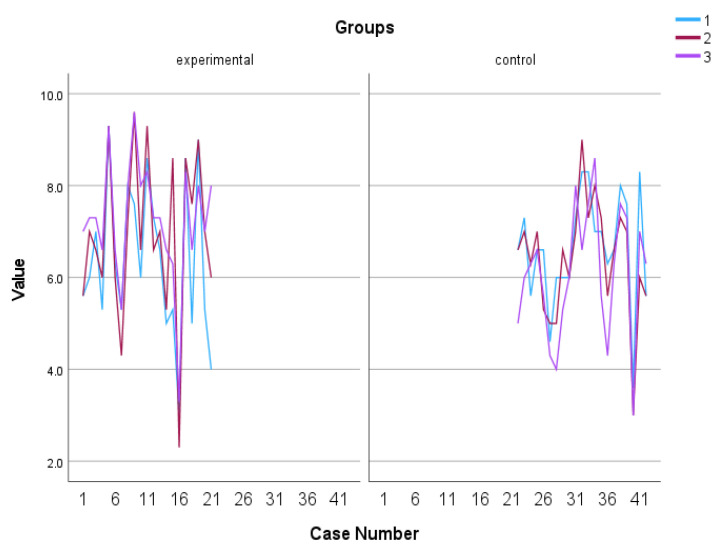
Individual scores in Engagement. Numbers 1, 2, and 3 explain the 3 assessment time points. Number 1 indicates before the PPI, number 2 indicates after the PPI and number 3 indicates the follow-up assessment.

**Figure 8:**
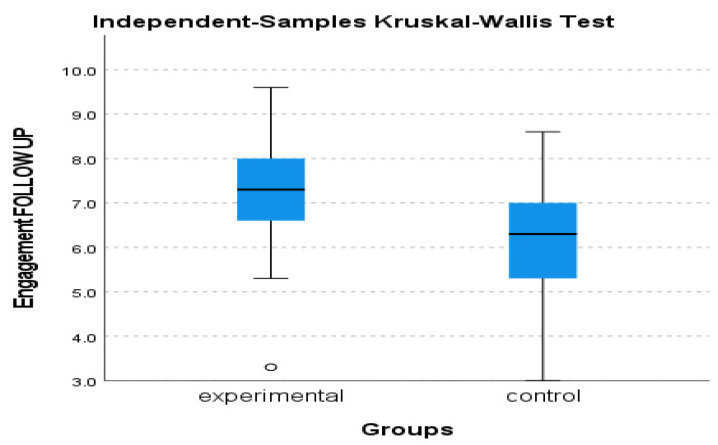
Engagement follow-up assessment of experimental and control group.

The utilization of a 2 (group) × 3 (time of assessment) mixed-measures ANOVA on the data regarding Engagement, as measured by the PERMA Profiler, revealed a statistically significant difference in group x time interaction, F (2, 39) = 9.406, *p* = 0.001, η^2^ = 0.325 with the experimental group tending to increase over time while the control group tending to gradually decrease (see Figure 6 and Figure 7). In addition, Kruskal–Wallis showed a significant difference between groups in terms of the Engagement follow-up assessment, η^2^ = 0.778 and *p* = 0.005 (see Figure 8).

#### 3.1.4. Meaning

**Figure 9:**
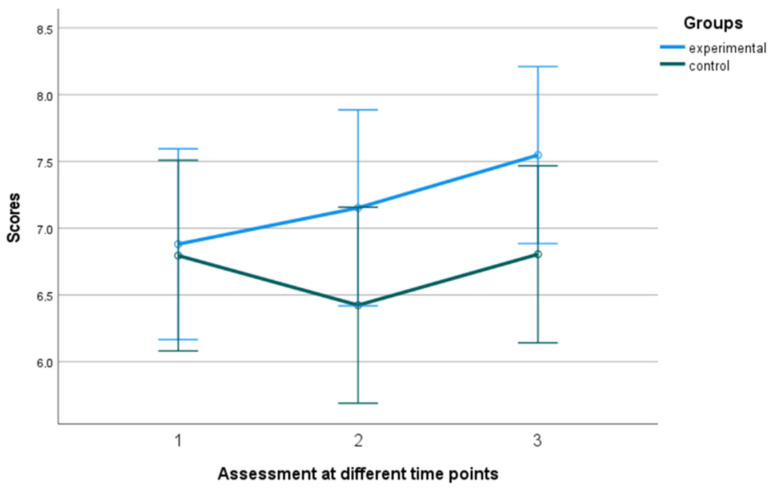
Performance of groups in Meaning in the 3 assessment time points. Numbers 1, 2, and 3 explain the 3 assessment time points. Number 1 indicates before the PPI, number 2 indicates after the PPI and number 3 indicates the follow-up assessment.

**Figure 10:**
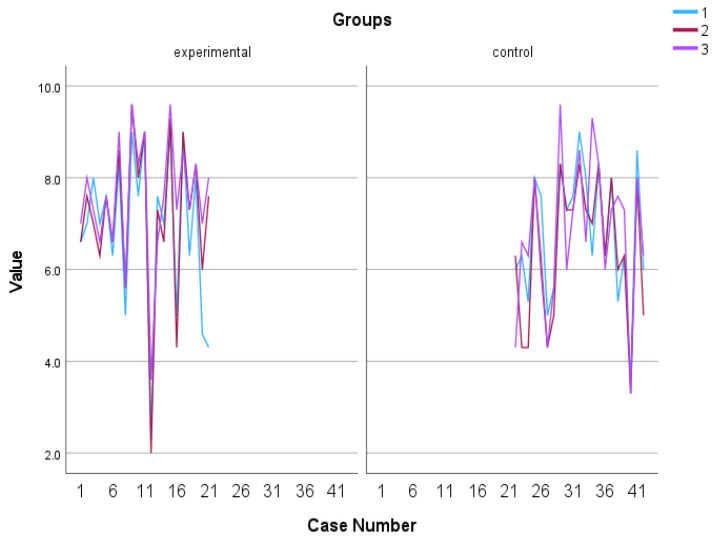
Individual scores in Meaning. Numbers 1, 2, and 3 explain the 3 assessment time points. Number 1 indicates before the PPI, number 2 indicates after the PPI and number 3 indicates the follow-up assessment.

To examine the impact of different assessment time points on Meaning, as measured by the PERMA Profiler, a 2 (group) × 3 (time of assessment) mixed-measures analysis of variance (ANOVA) was utilized where results revealed a significant interaction effect of time x intervention group, F (2, 39) = 3.479, *p* = 0.041, η^2^ = 0.151, as well as a significant main effect of time of assessment, F (2, 39) = 3.274, *p* = 0.048, η^2^ = 0.144. Both groups’ performances increased in the follow-up assessment when compared to the just after the intervention assessment, F (1,72, 39) = 3.912, *p* = 0.030, η^2^ = 0.089, I-J = -0.338, *p* = 0.040 (see Figure 9 and Figure 10).

#### 3.1.5. Accomplishment

**Figure 11:**
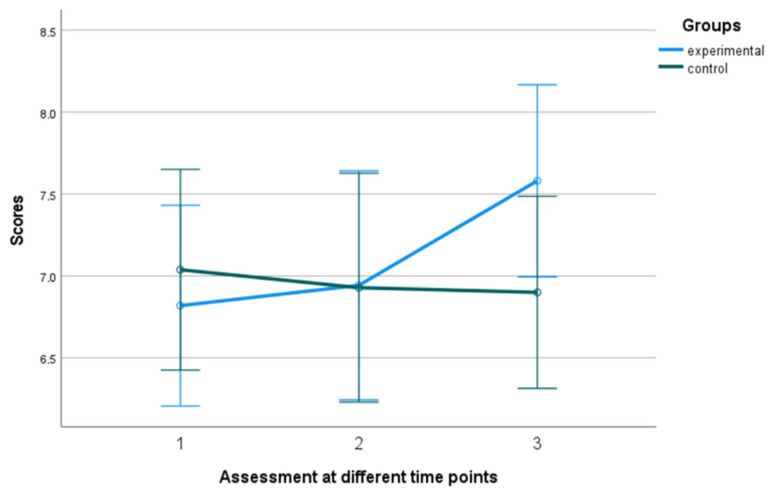
Performance of groups in Accomplishment in the 3 assessment time points. Numbers 1, 2, and 3 explain the 3 assessment time points. Number 1 indicates before the PPI, number 2 indicates after the PPI and number 3 indicates the follow-up assessment.

**Figure 12:**
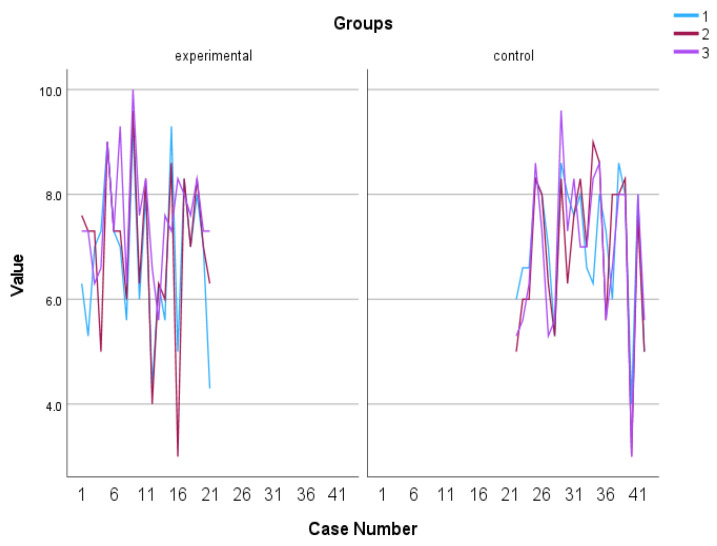
Individual scores in Accomplishment. Numbers 1, 2, and 3 explain the 3 assessment time points. Number 1 indicates before the PPI, number 2 indicates after the PPI and number 3 indicates the follow-up assessment.

Regarding Accomplishment, the same statistical analysis was followed where results revealed a tendency for a significant interaction effect, F (2, 39) = 3.162, *p* = 0.053, η^2^ = 0.140. The experimental group initially tended to exhibit lower scores compared to the control group. However, both groups tended to demonstrate a sharp increase in Accomplishment scores during the final assessment (see Figure 11 and Figure 12).

## 4. Discussion

This pilot study aimed to investigate the impact of a PPI on the subjective well-being of older individuals diagnosed with amnestic MCI. To achieve this, the study focused on 6 fundamental principles of positive psychology: gratitude, positive reminiscence, humor, kindness, and character strengths. Drawing from existing literature, these concepts were carefully defined, transformed into practical exercises, and implemented. The exercises aimed to redirect attention, memory, and expectations from negative aspects to positive aspects, thereby mitigating the adverse effects of negativity.

Overall, the findings of the study revealed that individuals who participated in the 3-week intervention exhibited higher scores in the overall well-being score as well as in all five dimensions of the PERMA model, in comparison to those who did not partake in the intervention. This confirms the first hypothesis of the study which stated that the PPI will increase the subjective well-being of amnestic MCI patients. This positive trend persisted even after one month following the completion of the intervention, confirming the second hypothesis of sustaining the increased subjective well-being for at least one month after the end of the PPI, and indicating a sustained impact on well-being. In addition, participants who did not engage in the intervention experienced a gradual decline in their performance across all domains, except for the domain of Positive Emotion, which showed a slight improvement. Interestingly, the domain of Meaning initially declined but demonstrated a recovery in scores during the follow-up period. 

It is important to mention some possible mechanisms behind the overall tendency for increased scores in all the variables considered in the study. Firstly, the interventions involved in this study may have helped participants develop a deeper sense of meaning and purpose in their lives. By reflecting on positive memories, expressing gratitude, engaging in acts of kindness, and leveraging personal strengths, individuals could find greater meaning and purpose in their daily experiences. 

Secondly, by exploring and utilizing character strengths, participants may have experienced personal growth, a sense of competence, and a greater alignment between their values and actions. This sense of growth and fulfillment could have contributed to overall well-being. Engaging in character strengths and positive reminiscence activities may have also led to developing a greater sense of resilience, allowing participants to navigate stressors more effectively and positively impact their well-being.

Additionally, the humor-based intervention utilized in the research may have functioned as a stress buffer, potentially alleviating negative emotions. Previous studies have established a correlation between humor and the release of endorphins, the reduction in stress hormones, and improvements in mood [44], all of which have the potential to enhance overall well-being. Humor is also claimed to promote amusement, a critical component of overall well-being, which can also operate as a defense against adverse conditions and fulfill other beneficial roles [45].

Moreover, positive reminiscence may enhance self-esteem [46], elicit enjoyment, and serve as a coping mechanism for negative emotions [47]. It facilitates the construction of a cohesive life narrative and fosters a sense of continuity in one’s personal history. Through reflection on positive memories, individuals could have integrated past experiences into a coherent framework, enhancing their understanding of personal identity and values. This sense of continuity in life story contributes to an enhanced sense of self and overall well-being. Furthermore, gratitude has a positive link with subjective well-being. Individuals who practice gratitude tend to be happier, have greater physical health, be more resilient, and participate in more prosocial behavior [48]. Moreover, as proven by Warneken (2018), acts of kindness evoke brain wave patterns similar to those linked with reward and pleasure [49]. In their cross-cultural study, Dunn et al. (2008) found that for example, people across the world are happier when they spend money on others rather than themselves [50].

The findings of this study have several important implications. Firstly, the findings align with previous PPIs, supporting the effectiveness of PPIs in enhancing subjective well-being [3,51], particularly among older adults [52]. The results highlight that PPIs can significantly improve the well-being of older individuals in the precursor stage of dementia. This is crucial as subjective well-being plays a key role in overall quality of life [53] and mental health [54].

Moreover, the study demonstrates the effectiveness of even a short-term 3-week intervention with a holistic approach in addressing multiple dimensions of well-being and generating lasting fulfillment and satisfaction. The sustained impact of the intervention, even after one month, underscores its significance in supporting individuals with amnestic MCI. The contrasting outcomes in the non-intervention group highlight the potential negative consequences of not engaging in well-being activities, while also indicating the possibility of limited positive changes without intervention. Overall, these findings enhance our understanding of the benefits of PPIs for older individuals with MCI. They emphasize the importance of targeting specific well-being domains and integrating such interventions into their care and support.

All in all, in future research it is important to consider realizing another PPI with an extended duration of at least five weeks, accompanied by a more extensive follow-up to allow a more comprehensive assessment of the possible long-term effects of the PPI. Moreover, to enhance the study’s validity, the sample should contain not only more participants in general but also more male participants with a diagnosis of MCI who are not part of any therapeutic program.

### Limitations and Future Research

This study’s findings should be interpreted in light of certain limitations. Firstly, the participants were exclusively recruited from Alzheimer’s Hellas, potentially limiting generalizability to individuals with amnestic MCI outside similar organizations. Future research should involve broader participant recruitment to ensure greater diversity among individuals with amnestic MCI.

Secondly, subjective well-being was measured only one month after the completion of the intervention. Long-term follow-up assessments would provide insights into the durability of the intervention’s effects [55]. Future research should include multiple follow-up assessments at different intervals to examine the long-term impact of the intervention on subjective well-being.

Thirdly, the study had a predominantly women sample (88.1%), which may introduce gender bias and restrict generalizability. Future research should strive for a more balanced gender representation and explore potential gender differences in the effectiveness of PPIs for individuals with amnestic MCI. However, it’s crucial to emphasize that the predominance of female patients in the Greek Alzheimer’s Association accurately represents the MCI patient demographic in this study. Lastly, the relatively small sample size in this study is a notable limitation, as it may limit the generalizability of the findings, reduce statistical power, and introduce the potential for selection bias. Increasing the sample size would enhance the statistical significance of the results, making them more robust and conclusive. 

## 5. Conclusions

In conclusion, this research aimed to explore the efficacy and PPIs in enhancing the subjective well-being of individuals with amnestic MCI. This pilot study found that the PPI indeed improved the subjective well-being of older individuals with amnestic MCI, supporting Hypothesis 1. Furthermore, the positive effects of the intervention were maintained one month after its completion (follow-up), also supporting Hypothesis 2. These findings suggest that PPIs hold promise in improving the well-being of individuals with MCI and have potential implications for addressing the growing burden of MCI and dementias in aging populations. Further research is needed to expand our understanding of the specific effects of PPIs on individuals with MCI and to develop targeted interventions to enhance their well-being.

## Figures and Tables

**Table 1:** Demographic characteristics of participants in experimental and control groups (n = 42).

Variables	Experimental	Control
	M (S.D.)	M (S.D.)
Age (in years)	71.62(4.3)	72.71(4.9)
Men % (n)	9.5% (2)	14.3% (3)
Women % (n)	90.5% (19)	85.7% (18)
Primary education (n)	14.3% (3)	9.5% (2)
Secondary education (n)	33.3% (7)	47.6% (10)
Higher education (n)	52.3% (11)	42.9% (9)

**Table 2:** Session-by-session overview of the PPI program for amnestic MCI patients.

Session	Themes	Purpose	Brief Description
1	Gratitude	The first session aimed to introduce the group members and learn about the benefits of gratitude. Cultivate gratitude, increase awareness of positive aspects of life, foster a positive outlook, enhance overall wellbeing, and develop a more optimistic perspective.	Three good things in life: Individuals were encouraged to reflect on and document for one week three positive events or experiences that occurred during their day, along with an explanation of why these events were meaningful or significant to them.
2	Gratitude	The second session aimed to better understand the concept of gratitude and how it can be put into practice. Increase feelings of appreciation, strengthen relationships, foster a sense of connection and wellbeing, experience an uplift in positive emotions, and develop a greater sense of gratitude towards others.	Gratitude visit: Individuals were encouraged to write a letter expressing gratitude to someone who has positively impacted their lives but has not been properly thanked. After composing the letter, individuals were also encouraged to arrange a meeting with the recipient and read the letter aloud, expressing their gratitude in person.
3	Positive reminiscence	In session three, the goal was to talk about the benefits of memories and how they can be used in our own favor. Emotional wellbeing, improved self-esteem, increased life satisfaction, and stress reduction are among its great benefits.	The magic box of memories: Individuals were told to each pick, write down, and read out loud one positive memory from their past. Based on what that memory was about, the other members of the subgroup were instructed to share a similar positive memory of their own. Participants were also requested to locate an object or item that serves as a reminder of their chosen positive experience.
4	Humor	The fourth session introduced the concept of humor and its benefits. Enhance positive emotions and overall wellbeing, reduces stress, increase social connections, foster positive relationships, improve communication, and promote a positive perspective, resilience, and coping skills.	Three funny things: Participants were asked to write down 3 funny things that happened to them during that day or the previous one. They were encouraged to recall the details of these funny experiences, relive the laughter or amusement they brought, and reflect on the positive emotions associated with them.
5	Kindness	In session number five, the goal was to talk about kindness and mention the benefits of the offer. Promote compassion, empathy, and prosocial behavior, contribute to the wellbeing of others, and experience increased positive emotions, a sense of fulfillment, and a strengthened sense of community.	Random acts of kindness: Participants were encouraged to first brainstorm and then perform for one-week acts of kindness, such as helping someone in need, offering a kind gesture, or performing small acts of generosity, without any expectation of reward or recognition.
6	Character strengths	In the last session, the subject was to identify and understand the positive qualities/strengths everyone carries and to close the intervention. Increase self-awareness, brings up a positive identity, and promote personal growth and development.	You at your best: Participants were asked to recall and reflect on a specific moment or experience in their life when they felt at their best, accomplished, or in a state of flow. It could be a personal achievement, a fulfilling project, or a time when he overcame a challenge. Then, they were asked to identify the personal strengths or qualities that they showed at that moment.

**Table 3:** Mean Scores and Standard Deviations of participants’ overall well-being and PERMA model dimensions across 3 assessment time points.

	Overall Wellbeing ^1^	Overall Wellbeing ^2^	Overall Wellbeing ^3^
Mean (S.D.)	Experimental 6.8 (1.3) Control 6.6 (1.3)	Experimental 7.2 (1.3) Control 6.6 (1.3)	Experimental 7.5 (1.0) Control 6.6 (1.3)
	Positive Emotion ^1^	Positive Emotion ^2^	Positive Emotion ^3^
Mean (S.D.)	Experimental 6.9 (1.6) Control 6.2 (1.9)	Experimental 7.4 (1.2) Control 6.4 (1.6)	Experimental 7.4 (1.2) Control 6.5 (1.3)
	Relationship ^1^	Relationship ^2^	Relationship ^3^
Mean (S.D.)	Experimental 7.2 (1.9) Control 6.7 (1.9)	Experimental 7.4 (1.8) Control 6.7 (1.5)	Experimental 7.7 (1.7) Control 6.8 (1.6)
	Engagement ^1^	Engagement ^2^	Engagement ^3^
Mean (S.D.)	Experimental 6.3 (1.6) Control 6.6 (1.2)	Experimental 6.9 (1.7) Control 6.4 (1.2)	Experimental 7.2 (1.3) Control 6.0 (1.4)
	Meaning ^1^	Meaning ^2^	Meaning ^3^
Mean (S.D.)	Experimental 6.8 (1.7) Control 6.7 (1.4)	Experimental 7.1 (1.7) Control 6.4 (1.5)	Experimental 7.5 (1.3) Control 6.8 (1.6)
	Accomplishment ^1^	Accomplishment ^2^	Accomplishment ^3^
Mean (S.D.)	Experimental 6.8 (1.5) Control 7.0 (1.2)	Experimental 6.9 (1.6) Control 6.9 (1.5)	Experimental 7.5 (1.0) Control 6.9 (1.5)

Numbers 1, 2, and 3 explain the 3 assessment time points. Number 1 indicates before the PPI, number 2 indicates after the PPI and number 3 indicates the follow-up assessment.

## Data Availability

Data is available upon duly justified request.

## Appendix A

**Table A1:** CONSORT guideline.

Section/Topic	Item No	Checklist Item	Reported on Page No
Title and abstract			
	1a	Identification as a randomized trial in the title	
	1b	Structured summary of trial design, methods, results, and conclusions (for specific guidance see CONSORT for abstracts)	
Introduction			
Background and objectives	2a	Scientific background and explanation of the rationale	
	2b	Specific objectives or hypotheses	
Methods			
Trial design	3a	Description of trial design (such as parallel, factorial) including allocation ratio	
	3b	Important changes to methods after trial commencement (such as eligibility criteria), with reasons	
Participants	4a	Eligibility criteria for participants	
	4b	Settings and locations where the data were collected	
Interventions	5	The interventions for each group with sufficient details to allow replication, including how and when they were actually administered	
Outcomes	6a	Completely defined pre-specified primary and secondary outcome measures, including how and when they were assessed	
	6b	Any changes to trial outcomes after the trial commenced, with reasons	
Sample size	7a	How sample size was determined	
	7b	When applicable, explanation of any interim analyses and stopping guidelines	
Randomisation:			
Sequence generation	8a	Method used to generate the random allocation sequence	
	8b	Type of randomisation; details of any restriction (such as blocking and block size)	
Allocation concealment mechanism	9	Mechanism used to implement the random allocation sequence (such as sequentially numbered containers), describing any steps taken to conceal the sequence until interventions were assigned	
Implementation	10	Who generated the random allocation sequence, who enrolled participants, and who assigned participants to interventions	
Blinding	11a	If done, who was blinded after assignment to interventions (for example, participants, care providers, those assessing outcomes) and how	
	11b	If relevant, description of the similarity of interventions	
Statistical methods	12a	Statistical methods used to compare groups for primary and secondary outcomes	
	12b	Methods for additional analyses, such as subgroup analyses and adjusted analyses	
Results			
Participant flow (a diagram is strongly recommended)	13a	For each group, the numbers of participants who were randomly assigned, received intended treatment, and were analysed for the primary outcome	
	13b	For each group, losses and exclusions after randomization, together with reasons	
Recruitment	14a	Dates defining the periods of recruitment and follow-up	
	14b	Why the trial ended or was stopped	
Baseline data	15	A table showing baseline demographic and clinical characteristics for each group	
Numbers analysed	16	For each group, number of participants (denominator) included in each analysis and whether the analysis was by original assigned groups	
Outcomes and estimation	17a	For each primary and secondary outcome, results for each group, and the estimated effect size and its precision (such as 95% confidence interval)	
	17b	For binary outcomes, presentation of both absolute and relative effect sizes is recommended	
Ancillary analyses	18	Results of any other analyses performed, including subgroup analyses and adjusted analyses, distinguishing pre-specified from exploratory	
Harms	19	All important harms or unintended effects in each group (for specific guidance see CONSORT for harms)	
Discussion			
Limitations	20	Trial limitations, addressing sources of potential bias, imprecision, and, if relevant, multiplicity of analyses	
Generalisability	21	Generalisability (external validity, applicability) of the trial findings	
Interpretation	22	Interpretation consistent with results, balancing benefits and harms, and considering other relevant evidence	
		Other information	
Registration	23	Registration number and name of trial registry	
Protocol	24	Where the full trial protocol can be accessed, if available	
Funding	25	Sources of funding and other support (such as supply of drugs), role of funders	
